# Multi-segment rupture of the 2016 Amatrice-Visso-Norcia seismic sequence (central Italy) constrained by the first high-quality catalog of Early Aftershocks

**DOI:** 10.1038/s41598-019-43393-2

**Published:** 2019-05-06

**Authors:** Luigi Improta, Diana Latorre, Lucia Margheriti, Anna Nardi, Alessandro Marchetti, Anna Maria Lombardi, Barbara Castello, Fabio Villani, Maria Grazia Ciaccio, Francesco Mariano Mele, Milena Moretti, The Bollettino Sismico Italiano Working Group, P. Battelli, M. Berardi, C. Castellano, C. Melorio, G. Modica, M. Pirro, A. Rossi, C. Thermes, N. Pagliuca, S. Spadoni, L. Arcoraci, A. Battelli, A. Lisi, L. Pizzino, P. Baccheschi, B. Cantucci, A. Sciarra, A. Bono, C. Marcocci, V. Lauciani, A. Mandiello, S. Pintore, M. Quintiliani, A. Frepoli, L. Colini, S. Pinzi, L. Scognamiglio, A. Basili, G. D’Addezio, T. Sgroi, A. Smedile, C. Montuori, R. Tardini, R. Tozzi, S. Monna, L. Miconi, M. T. Mariucci, R. Di Maro

**Affiliations:** 1 Istituto Nazionale di Geofisica e Vulcanologia, Osservatorio Nazionale Terremoti, Roma, Italy; 2 Istituto Nazionale di Geofisica e Vulcanologia, Sezione Roma 1, Roma, Italy; 3 Istituto Nazionale di Geofisica e Vulcanologia, Sezione Roma 2, Roma, Italy

**Keywords:** Tectonics, Seismology

## Abstract

We present the first high-quality catalog of early aftershocks of the three mainshocks of the 2016 central Italy Amatrice-Visso-Norcia normal faulting sequence. We located 10,574 manually picked aftershocks with a robust probabilistic, non-linear method achieving a significant improvement in the solution accuracy and magnitude completeness with respect to previous studies. Aftershock distribution and relocated mainshocks give insight into the complex architecture of major causative and subsidiary faults, thus providing crucial constraints on multi-segment rupture models. We document reactivation and kinematic inversion of a WNW-dipping listric structure, referable to the inherited Mts Sibillini Thrust (MST) that controlled segmentation of the causative normal faults. Spatial partitioning of aftershocks evidences that the MST lateral ramp had a dual control on rupture propagation, behaving as a barrier for the Amatrice and Visso mainshocks, and later as an asperity for the Norcia mainshock. We hypothesize that the Visso mainshock re-activated also the deep part of an optimally oriented preexisting thrust. Aftershock patterns reveal that the Amatrice Mw5.4 aftershock and the Norcia mainshock ruptured two distinct antithetic faults 3–4 km apart. Therefore, our results suggest to consider both the MST cross structure and the subsidiary antithetic fault in the finite-fault source modelling of the Norcia earthquake.

## Introduction

The key role of geometrical fault complexity in controlling both earthquake nucleation and dynamic rupture propagation is a key issue in the seismic source mechanics^1^, and has been evidenced by numerous multi-fault rupture earthquakes^2^. Among recent earthquakes, the 2010 Mw7.2 El Mayor-Cucapah (Mexico) and the 2016 Mw7.8 Kaikoura (New Zealand) events are illuminating examples of rupture cascades involving distinct fault segments with variable orientations and kinematics^3,4^. While there exists a wealth of examples of multi-fault large earthquakes for strike-slip and transpressional settings^2^, the literature is quite limited for normal-faulting events. The large stress perturbation associated with the 2011 M9.0 Tohoku-Oki mega-thrust earthquake is believed to have induced the sequence of M6-7 normal faulting earthquakes that ruptured complex arrays of fault segments along the Pacific coast of NE Japan^5^. Extensional provinces affected by multi-fault normal-faulting earthquakes or sequences are: the Basin and Range in western North America^6^; the Afar in the East Africa Rift zone^7^; the North Island of New Zealand^8^; the continental Greece^9,10^; the western Cordillera Belt in Spain^11^; the Apennine seismic belt of Italy^12^. For the Apennines, multi-fault ruptures during a single seismic event (e.g. 1980 Ms 6.9 Irpinia-Basilicata)^13^ or sequences spanning a period of days (e.g. 2009 Mw 6.1 L’Aquila)^14^ to months (e.g. 1997 Mw 6.0 Colfiorito)^15^, are controlled by the inherent geometrical complexity of the active normal fault systems dissecting a mountain belt mainly made of carbonate and siliciclastic rocks, and characterized by a poly-phased tectonic evolution. Extensional tectonics took place since the Early and Middle Pleistocene in central^16^ and southern Apennines^17^, replacing the Neogene compressional tectonics responsible for the thrust-belt accretion and overprinting contractional structures. The average Quaternary extension axis trends NE-SW, mostly parallel to the direction of the orogenic transport, so that several normal faults are nearly coaxial with thrusts. The relative youthfulness of the extension regime and the heterogeneity of the sedimentary multi-layer involved in the orogenic wedge played a key role in the segmentation of the NW-SE trending active normal fault systems^16^. As a consequence, there is growing evidence that pre-existing compressional structures may control rupture nucleation and propagation during multi-fault normal-faulting earthquakes and seismic sequences. Crustal-scale heterogeneities (e.g., wide ramp anticlines formed by high-strength carbonates), arcuate thrust faults and transpressive cross-faults often limit the extent of individual ruptured segments^15,18^, while pre-existing thrust faults optimally oriented to slip in the present-day extensional stress field (i.e., NW-SE trending) can be in turn reactivated as normal faults under proper dynamic conditions^18–20^. For the Apennines, the extensional reactivation of favorably oriented Pliocene thrust faults is also documented by detailed analysis of fluid-injection induced seismicity^21^.

The Amatrice-Visso-Norcia (AVN) seismic sequence that struck central Apennines with three mainshocks with normal-faulting mechanisms between August and October 2016 poses significant challenges from the perspective of earthquake source characterization and analysis of seismic sequence evolution. In particular, the geometry of the activated normal faults and preexisting compressional structures has emerged as a central element of the scientific discussion about the multi-segment rupture process.

**Figure 1:**
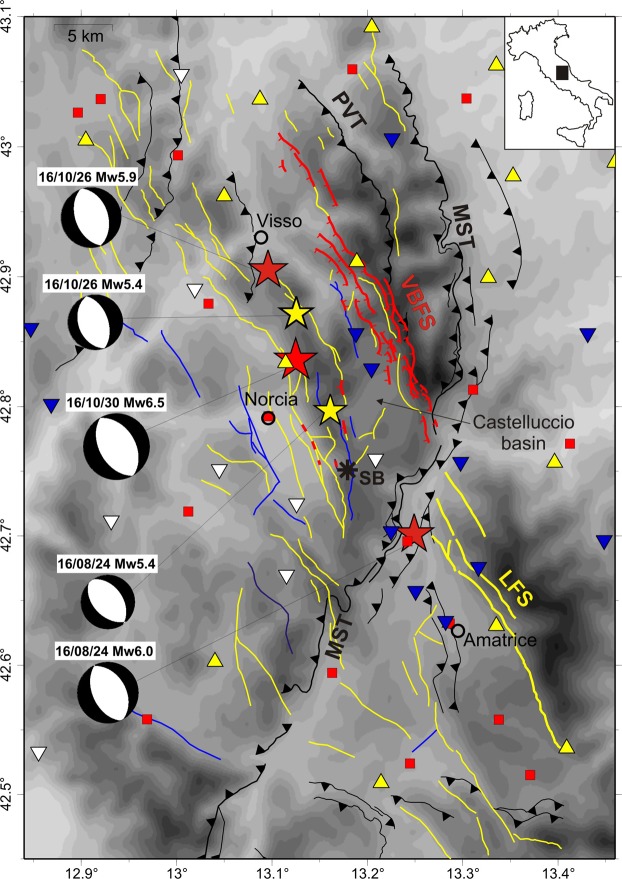
Map of the epicentral area showing main active normal faults. Yellow lines denote W-dipping fault, blue lines E-dipping faults, yellow solid lines the Laga Fault system (LFS), red solid lines co-seismic surface ruptures along the Vettore-Bove fault system (VBFS) (modified after)^41^. The black barbed lines are Miocene-Pliocene thrusts (MST: Mts Sibillini Thrust; PVT: Pizzo Tre Vescovi thrust). Red stars denote the three mainshocks and yellow stars M5+ events, with associated TDMT focal mechanisms (http://cnt.rm.ingv.it/tdmt). Yellow triangles are stations of the INGV National Seismic Network, blue triangles real-time temporary stations, white triangles temporary stations used for the first time in this study. Red squares denote DPC accelerometric stations used to re-locate the mainshocks and M5+ events. Black asterisk (label *SB*) indicates the coseismic faulting that affected the San Benedetto freeway tunnel.

The sequence initiated on 24 August 2016 with the Mw 6.0 Amatrice earthquake^22,23^ and was followed on 26 October 2016 by the Mw 5.9 Visso earthquake, ∼25 km to the north (Fig. 1). The largest event, the Mw 6.5 Norcia earthquake, occurred on 30 October and nucleated in between the source regions of the two previous mainshocks. Each mainshock was followed by sustained aftershock activity, and in the following months seismicity migrated southward and northward. The activated zone is 70-km-long and 10-km-thick, and trends NNW-SSE parallel to the axis of the central-northern Apennines.

Moment tensor solutions for the mainshocks, InSAR images, primary surface faulting, and aftershock distribution, point consistently to normal-faulting along NNW-SSE trending and WSW-dipping faults referable to two sub-parallel Quaternary systems, the Mt.Vettore-Mt. Bove fault system to the north (VBFS) and the Laga Fault system to the south^24^ (Fig. 1).

Finite-fault inversions of the mainshocks using strong-motion and/or geodetic data evidence multi-fault ruptures on adjacent segments of the two normal fault systems^23–28^. Fault segmentation is ascribed to the inherited Mio-Pliocene Mts Sibillini Thrust (MST, Fig. 1), whose NNE trending lateral ramp in turn possibly ruptured during the Norcia mainshock^29^. Aftershock clusters in the footwall of the VBFS suggest the reactivation of inherited reverse faults^22^.

Seismic reflection data published so far are limited to a regional section merging three commercial profiles of fair to low resolution that crosses the epicentral area and the Vettore Fault^30^. Thus, main inferences on the deep architecture of the fault systems activated during the whole sequence come primarily from aftershock distribution^22,31^.

Aftershocks catalog published by^22^ contains 25,600 events for the period between August 24 and November 29 2016 re-located by inverting first-arrival times handpicked routinely in quasi real-time at the surveillance center of Istituto Nazionale di Geofisica e Vulcanologia (INGV). Data were recorded by the INGV National Seismic Network complemented by 12 temporary stations installed soon after the Amatrice earthquake and connected in real-time to improve performance of the monitoring system^32^. Notwithstanding the good quality of this dataset, the catalog published by^22^ suffers from implicit drawbacks strictly related to the fact that the analysis procedure at the INGV surveillance center must provide very fast and reliable hypocentral location and local magnitude estimation for civil protection purposes (manually revised parameters are commonly released in 15–20 minutes). The location procedure routinely followed during a seismic sequence is based on the revision of P-and S-phase automatic picks provided by the earthquake detection system, complemented by hand-picking of P- and S-arrivals for a limited number of stations surrounding the real-time automatic location. Additionally, in the first days following the AVN mainshocks, seismologists on duty at the surveillance center had to focus just on the strongest aftershocks. Indeed, revision of small early aftershocks detected by the monitoring system posed significant challenges because of the occurrence of very close or even simultaneous events located in different zones of the monitored region.

From the previous considerations, it arises that the catalog published by^22^ contains only a subset of the aftershocks detected by the real-time monitoring system, with the number of missed events being larger for early aftershocks, and that events re-location uses only part of the stations and readable arrival times.

The enhanced aftershock production over a wide zone also makes the use of automatic phase picker tools difficult. The local earthquake tomography survey of^31^, which uses automatic traveltime pickings of ∼44,100 events recorded from 24 August 2016 to June 2017, shows a relatively-low spatial resolution (5 km horizontally and 3 km vertically) and variance improvement (32%) in spite of the good station source-receiver geometry, suggesting that uncertainty in the arrival time data degraded the inversion process.

To overcome the previous drawbacks, we performed a careful reprocessing of all the earthquakes detected by the INGV real-time monitoring system in the first days after the Amatrice, Visso and Norcia earthquakes to create the first high-quality and comprehensive catalog of early aftershocks. P- and S-wave first arrivals were hand-picked using all INGV stations installed in the epicentral region and inverted by using a robust, non-linear earthquake location algorithm^33^. We decided to focus our analysis on early aftershocks for two reasons: first, the number of events detected by the real-time monitoring system but not revised manually was the largest in the first hours after moderate-strong shocks of the sequence; second, accurate locations of early aftershocks can give clues on the initial fault activation and on the geometry and size of mainshock faults providing key information to understand the source rupture processes. The contribution of early aftershocks can be fundamental in case of a multi-fault rupture involving segments with different orientations and kinematics during a single seismic event^3^. In addition, the anticorrelation between distribution of early aftershocks and main patches of slip can give insight into the rupture process and the pattern of asperities and barriers^1^.

The outcome is a high-quality catalog of 10,574 aftershocks characterized by a significant improvement in location accuracy and magnitude completeness with respect to previous studies. The new catalog provides a clearer image of the early aftershock seismicity that improves our understanding of the activated fault systems, thereby highlighting strength and weakness of multi-fault rupture models. In addition, our catalog is valuable for future seismological applications.

## Data Analysis and Earthquake Catalog

We analyzed all the aftershocks detected between August 24^th^–26^th^ (hereinafter early aftershocks of the Amatrice earthquake), October 26^th^–27^th^ (early aftershocks of the Visso earthquake) and October 30^th^–November 1^st^ 2016 (early aftershocks of the Norcia earthquake). We handpicked around 291,800 P- and 188,200 S-wave first arrivals of 10,574 aftershocks using 129 permanent and temporary INGV stations installed in the epicentral area (Fig. 1) and surrounding regions. Our thorough analysis is based on the: *(i)* revision of P- and S-phase available data (arrival times, data weights) of aftershocks quickly examined at the INGV surveillance center or simply detected by the real-time monitoring system, *(ii)* addition of P- and S-phase picks for numerous permanent and temporary stations that were not included in previous automatic or manually revised locations. In addition, our study benefits from data of 9 temporary INGV stations^32^, which are used for the first time.

We located events in a 1-D local velocity model by applying the non-linear inversion technique NonLinLoc^33^.

We computed local magnitude after a careful assessment of the quality of the amplitude measurements, aimed at both correctly managing records affected by the superposition of aftershocks and discarding stations affected by local site effects. Aftershocks have magnitude in the range 0.2–4.7.

With respect to data revised routinely at the INGV surveillance center, which were used by^22^, the new catalog shows a significant increase in the number of earthquakes (2.8 to 3.9 times larger depending on the mainshock) and improvement in magnitude completeness (Mc) (see Supplementary Fig. S1). Mc improves from 2.7 to 2.2 for aftershocks of the Amatrice earthquake, from 2.4 to 2.2 for Visso earthquake, and from 3.4 to 2.9 for the Norcia earthquake. The higher magnitude of completeness of the Norcia earthquake catalog is due to the significant aftershock production and enlargement of the activated zone that hamper analysis of small (M < 3) aftershocks due to the close occurrence of events with overlapping waveforms in the data streams.

The distribution of location quality parameters demonstrates the high-quality of the catalog (see Supplementary Fig. S2): 80% of hypocentral locations has *rms* ≤ 0.12 s, azimuthal gap ≤89°, horizontal and vertical formal location errors (2σ) ≤0.6 km and ≤2 km, respectively.

The comparison of quality parameters for event locations determined by inverting our revised data and those picked in quasi real-time at the INGV surveillance center, which were also used by^22^, evidences a remarkable improvement in location accuracy (see Supplementary Materials). The strong increase of the phase picks number for most aftershocks causes an important reduction of the azimuthal gap and epicentral distance to the closest station that primarily control horizontal and vertical location uncertainties, respectively (Supplementary Fig. S3). As a result, the percent of locations having horizontal formal location errors <0.6 km increases from 36% to 78%, while the percent of locations having vertical formal location errors <1.5 km increases from 27% to 71%. In particular, the revised data allow to better constraining hypocentral depth of shallow earthquakes.

To obtain accurate hypocenter locations for all M5+ shocks, we made a careful revision of strong motion recordings of permanent and temporary INGV stations, complemented by 11 to 19 stations of the Accelerometric National Network run by Department of Civil Protection (Fig. 1). With respect to previous catalogues^22,31^, our re-location provides significantly shallower hypocentral depth (Δz > 1 km) for all events but the Norcia mainshock (Supplementary Table S1). The larger mismatch (Δz = 3.4 km) attains to the Amatrice mainshock. We also used the same inversion procedure to relocate the hereafter called late aftershocks, i.e. earthquakes detected by the INGV monitoring center in the temporal intervals not analyzed in this study (49,544 events from 24 August 2016 to 24 July 2018).

## Results

### Aftershock distribution and fault-system geometry

The AVN sequence struck an area that spans two major structural domains, the Umbria-Marche thrust belt and the Laga foredeep domain, at the junction between central and northern Apennines^34^ (among others). The structural architecture of the range is very complex, because it derives from three main extensional and compressive tectonic pulses: (1) pre-orogenic syn-sedimentary extension (Jurassic) controlled the formation of pelagic basin and carbonate platform domains delimited by crustal-scale fault zones; (2) Mio-Pliocene compression affected the previously formed basin and platform domains and was responsible for the accretion of the Umbria-Marche belt structured into NE-verging arcuate thrust faults and associated folds; (3) Quaternary post-orogenic extension accommodated by NNW-SSE trending SW-dipping normal fault systems that dissect the fold-thrust belt and bound large intramontane depressions. The crustal architecture is dominated by the arcuate MST (Fig. 1) that juxtaposes the Meso-Cenozoic carbonate multilayer of the Umbria-Marche succession onto Miocene foredeep basin deposits of the Laga domain. In the MST hanging-wall, the Umbria-Marche sequence mainly consists of slope-basinal limestones (Jurassic-Oligocene), platform carbonates (Triassic-Jurassic), evaporites (dolostones and anhydrites, Triassic) and covers at about 9 km depth a Permo-Triassic phyllitic basement that in turn overlies crystalline units^35^. The orientation of the MST surface trace rotates from NNW in the northern part, to N and NNE in the central and southern parts, respectively (Fig. 1). The NNE-striking southern segment was interpreted as a transpressive lateral ramp reactivating a regional-scale fault zone of the Jurassic extension^36^, and it is oblique to the Quaternary normal fault systems. In particular, the NNE trending SW-dipping VBFS and Laga Fault system that are the causative faults of the AVN mainshocks, as well as of Holocene and historical M6+ earthquakes^37^, are located in the hangingwall and footwall of the MST, respectively (Fig. 1), and their growth, development and segmentation were clearly controlled by this inherited cross structure^16^. The complex interplay between Miocene-Pliocene thrust faults with associated anticlines and the NNW-SSE trending synthetic and antithetic normal faults that bound intermontane depressions, such as the Norcia and Castelluccio basins (Fig. 1), is documented by structural field data^38,39^ and recent geophysical surveys^40^.

The distribution of early aftershocks and mainshocks, complemented by relocated late aftershocks, yields valuable information on faults activated during the seismic sequence. Coherently with^22^, we recognized the following main trends of the aftershock distribution: *(i)* NNW-trending, SW-dipping event zones related to the causative faults of the three mainshocks, *(ii)* NNW-trending, NE-dipping event zones referable to antithetic normal faults, *(iii)* deep seismicity clustering at 8–10 km depth within a band gently dipping eastward.

**Figure 2:**
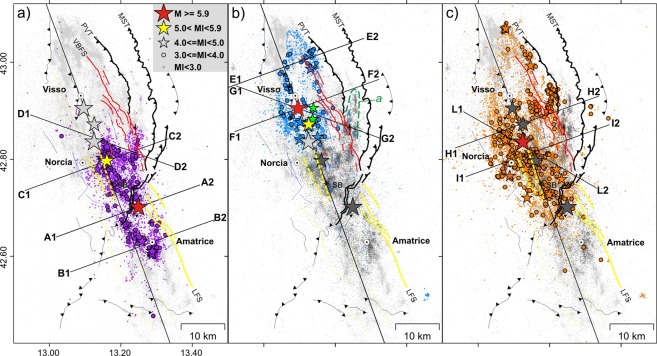
Map view of the relocated aftershocks. (**a**) Early aftershocks of the Amatrice earthquake (purple, August 24–26), (**b**) of the Visso earthquake (cyan, October 26–27) and (**c**) of the Norcia earthquake (orange, October 30–November 1). Grey dots are late aftershocks analyzed routinely in quasi real-time at the INGV surveillance center and relocated in this study (light and dark grey dots denote earthquakes occurred after and before the time interval considered in each panel, respectively). Colored lines are fault traces, with main faults ruptured during the seismic sequence evidenced by thick lines (fault symbols as in Fig. 1). Red solid lines correspond to co-seismic surface ruptures along the Vettore-Bove fault system (VBFS). Dashed thick yellow lines denote the up-dip projection (top at sea level) of the two antithetic normal faults defined in this study, ruptured by the Amatrice Mw5.4 aftershock (western fault) and the Norcia mainshock (eastern fault). Dashed green line in panel (b) delimits the aftershock cloud elongated N-S parallel to the MST frontal ramp (outlined by label *a*). Black asterisk (label *SB*) indicates the coseismic faulting that affected the San Benedetto freeway tunnel.

**Figure 3:**
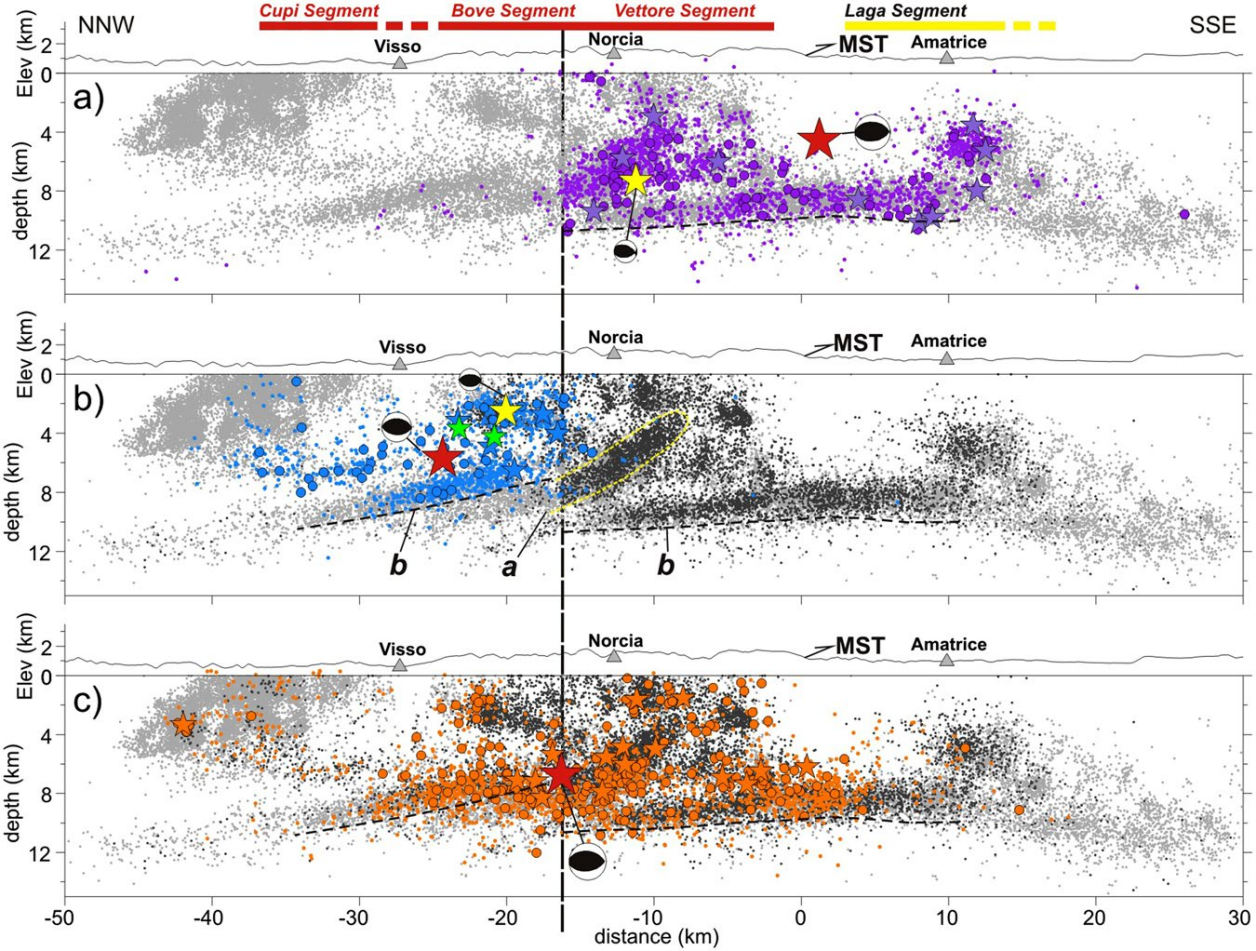
Aftershocks projected onto the N160°-oriented section showed in Fig. 2. (**a**) Aftershocks of the Amatrice earthquake, (**b**) of the Visso earthquake and (**c**) of the Norcia earthquake (symbols as in Fig. 2a–c). Hypocenters within 5 km distance from section are projected. Fault segments ruptured by the mainshocks are schematically reported on top of panel (a). The yellow dashed line (label *a*) outlines the WNW-dipping cloud of aftershocks of the Amatrice and Norcia earthquakes that relates to the MST lateral ramp. The black dashed lines define the bottom of the deep band of high aftershock production (label *b*).

**Figure 4:**
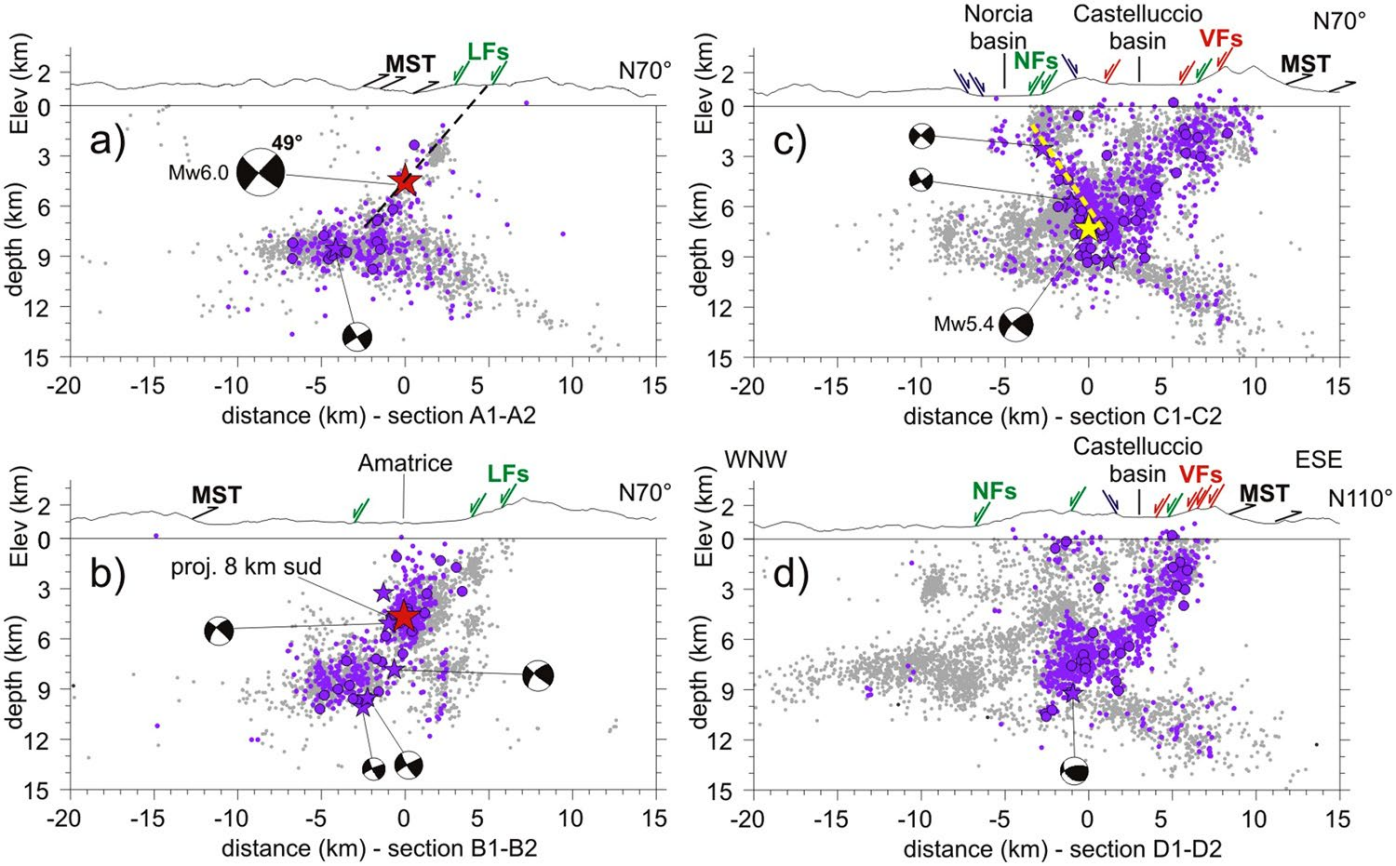
Early aftershocks of the Amatrice earthquake projected onto sections oriented orthogonal to the Laga Fault system (**a**,**b**), Vettore Fault (**c**) and Mts Sibillini Thrust (**d**). Light grey dots are aftershocks occurred after the considered time interval (24–26 August). Focal mechanisms are projected TDMT solutions of M > 4 events. The dashed yellow lines define fault planes delineated by aftershock alignments, the black dashed lines mainshock fault planes inferred by TDMT solutions. Hypocenters within 3 km distance from sections are projected. Red arrows outline surface rupturing segments of the Vettore Fault (VFs), green arrows W-dipping active normal faults (LFs - Laga Fault system, NFs - Norcia basin fault-system), blue arrow NE-dipping active normal faults, black arrows traces of the Mts Sibillini Thrust (MST).

**Figure 5:**
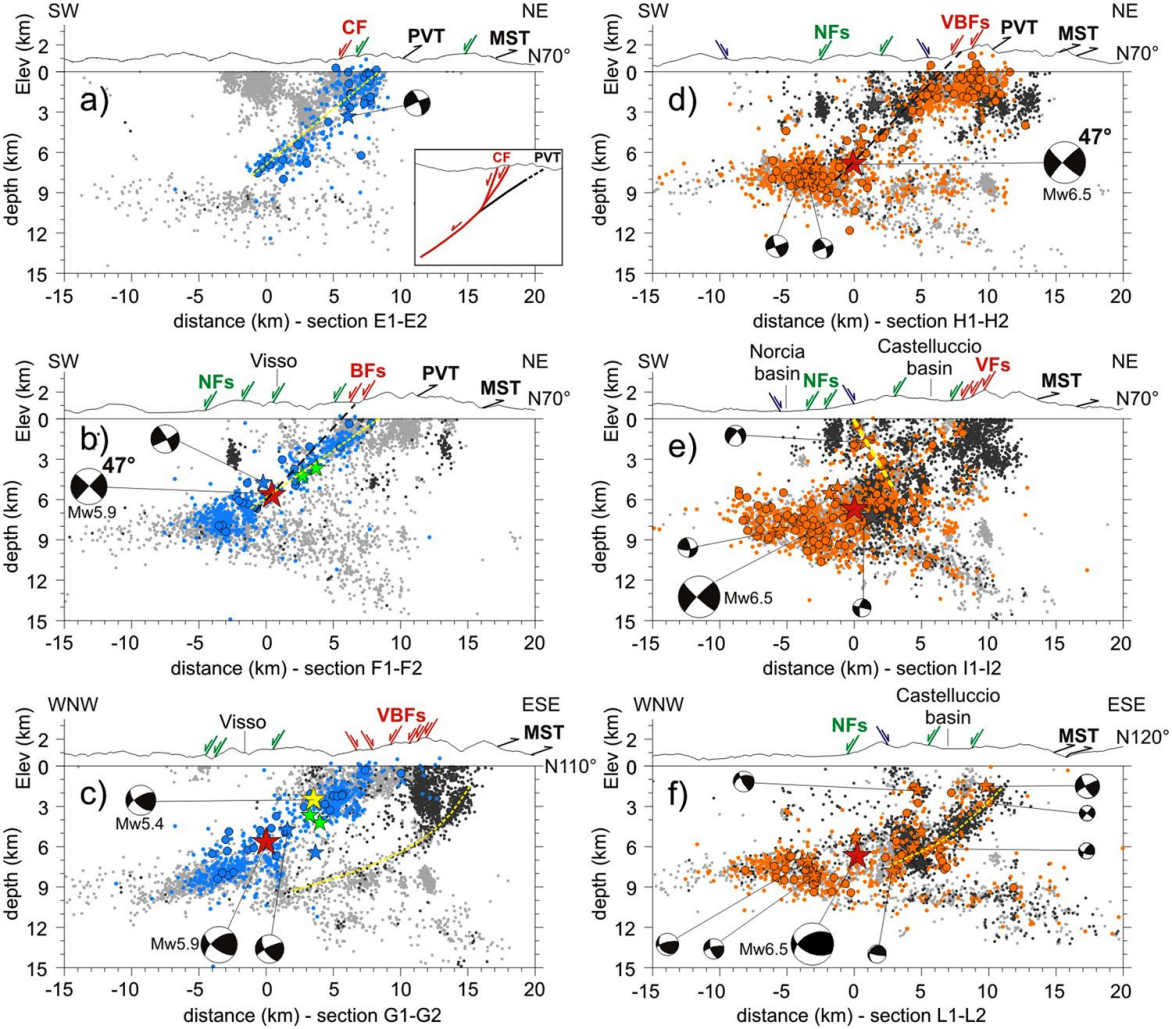
Early aftershocks of the Visso earthquake and Norcia earthquakes are projected onto sections oriented orthogonal to the Vettore-Bove fault system (**a**,**b** and **d**,**e**, respectively) and Mts Sibillini Thrust (**c**,**f**, respectively). Light and dark grey dots denote earthquakes occurred after and before the time interval considered in each panel, respectively. Focal mechanisms are projected TDMT solutions of M > 4 events. The dashed yellow lines define fault planes delineated by aftershock alignments, the black dashed lines mainshock fault planes inferred by TDMT solutions. Hypocenters within 3 km distance from sections are projected, with the exception of section *L1-L2* in panel (f) for which events are within 1.5 km distance. Red arrows outline surface rupturing segments of the of the Vettore-Bove fault system (VFs - Vettore Fault, BF - Bove fault, CF - Cupi Fault), green arrows W-dipping active normal faults (NFs - Norcia basin fault-system), blue arrow NE-dipping active normal faults, black arrows inherited thrusts (MST - Mts Sibillini Thrust, PVT - Pizzo Tre Vescovi thrust). The inset sketch of panel a illustrates the model hypothesized for the northern segment ruptured by the Visso earthquake (red lines define ruptured segments, the black line the shallow part of the Pizzo Tre Vescovi thrust prolific in off-fault aftershock production).

We analyzed the seismicity pattern and the fault-plane solutions from Time-Domain Moment Tensor (TDMT) inversion of the largest events (http://cnt.rm.ingv.it/tdmt) separately for the three investigated time intervals and compared them to precise surface constraints on Quaternary normal faults^41^ and Mio-Pliocene thrusts^38,42^ (Fig. 2a–c). Hereafter we describe 80-km-long longitudinal sections (N160°; Fig. 3a–c) to provide a general overview of the zones activated by each mainshock, together with cross-sections oriented N70°E (orthogonal to the Laga Fault system and VBFS) and N110–120°E (orthogonal to the MST), which show seismicity patterns related to first-order structures (Figs 4a–d and 5a–f). A 3-D perspective of the early and late aftershocks of the Amatrice, Visso and Norcia earthquakes, together with the planar sources obtained by finite-fault inversion of the three mainshocks^22,23,29^, aids in the understanding of the complex spatial distribution of seismicity (see Supplementary Videos S1, S2 and S3).

### Aftershocks of the Amatrice earthquake

The Amatrice earthquake ruptured two distinct shallow (<6 km depth) asperities^23^ for a total length of ∼25 km. The southern asperity correlates to the northern segment of the Laga Fault system, the northern one to the Vettore Fault (Fig. 3a).

Remarkably, the fault plane inferred by TDMT tied to our mainshock hypocenter perfectly fits the eastern splay of the Laga Fault system (Fig. 4a). A wide zone surrounding the mainshock hypocenter is devoid of aftershocks (Figs 2a, 3a and 4a), which concentrate northward and southward at the edges of the asperities resolved by finite-fault inversions (Figs 2a, 3a and 4b,c). To the south, distribution of early aftershocks follows a SW-dipping trend coherent with the mainshock focal solution (Fig. 4b). Only a few, sparse M_L_ < 3 events are located in the top 2 km of the crust in agreement with the lack of evident surface faulting along the Laga Fault system^41^ and the 2 km thick cover of marly and terrigenous Miocene sediments.

To the north, the aftershock distribution delineates two oppositely dipping structures (Fig. 4c and Supplementary Video S1). The eastern one dips SW and a part of shallow aftershocks correlates with the Vettore Fault. Differently from the Laga Fault system, the Vettore segment appears associated with shallow aftershocks (M3+) in accordance with shallow slip required by finite-fault inversion models^28^ and surface coseismic faulting observed for ∼6 km in strong Mesozoic carbonate rocks^39^ (maximum surface slip of 0.35 m). The easternmost shallow aftershocks fall in the Vettore Fault footwall delimited by surface ruptures (Fig. 4c). In map view, those aftershocks define a N-trending cloud (Fig. 2a) that elongated further northward for more than 10 km beyond the Vettore Fault in the following weeks (Fig. 2b, label *a*). Their hypocenters projected onto a N110° section (i.e. orthogonal to the thrust fronts) collapse to a WNW-dipping narrow band (Fig. 4d): the dip angle is ~50° in the upper part, tending to decrease with depth. Since the cloud of epicenters of off-fault aftershocks follows the curvature of the MST (Fig. 2b, label *a*) and the surface projection of event alignment is compatible with mapped thrust traces (Fig. 4d), we conclude that aftershocks of the Amatrice earthquake re-activated, as a normal fault, a high-angle structure in the footwall of the VBFS likely related to the eastern inherited thrust (Fig. 2a,b). However, at the southern termination of the VBFS where the Vettore fault and the MST are very close, aftershocks do not allow to discriminate between the two faults (as showed in the next paragraph, the northern sections delineate instead two distinct structures).

The western alignment of early aftershocks delineates a plane dipping ∼55°NE (Fig. 4c and Supplementary Video S1). Aftershock distribution and TDMT of M4+ events suggest that the August 24 Mw5.4 aftershock ruptured an antithetic normal fault. Fault strike and length inferred from event alignment are ∼N330° and ∼10 km, respectively (Fig. 2a). This structure is buried under the eastern side of the Norcia basin and parallel to the western basin-bounding fault (Fig. 1).

### Aftershocks of the Visso earthquake

The Visso earthquake ruptured the northern and central segments of the VBFS^24^ for a length of ∼20 km (Cupi and Bove faults, respectively; Fig. 3b).

Our analysis reveals that the Visso earthquake and its strong foreshock (Mw5.4) are double earthquakes, both preceded by M_L_4.5 events at 4.0 and 1.5 km distance, respectively (green stars in Figs 2b and 3b).

If we shift TDMT solution of the mainshock to our hypocentral location, the surface projection of the 47°-dipping plane would match the primary coseismic ruptures observed for a minimal length of 7 km with average surface slip of 0.11 m^39^ (Fig. 5b). However, such a simple model of a planar fault does not reconcile with the aftershock distribution because early aftershocks align on a lower-angle plane (∼35°), while shallow events (<2 km depth) fall mostly to the east of the surface rupturing segments, that is in their footwall (Fig. 5a,b). These findings suggest a different model implying the involvement of a structure located between the VBFS and MST. Based on available surface geological data^42^, we do not have conclusive elements to relate such low-angle aftershock alignment to a known structure: as best candidate, we propose the deeper part of the Pizzo Tre Vescovi thrust (Fig. 1, label PVT). The latter is an arcuate and >18 km-long structure in the hanging-wall of the MST exposed along the eastern flank of the Sibillini Mts. Indeed, the surface projection of the structure delineated by early aftershocks matches the thrust trace (Fig. 5a,b), while the epicentral distribution of shallow aftershocks follows its strike, rotating from NW in the northern part to NNW in the southern one (Fig. 2b).

We propose that the Visso earthquake rupture first propagated up-dip onto the deeper portion of the thrust and subsequently onto the shallow and steeper splays of the Cupi fault system, which caused surface faulting (see the sketch in Fig. 5a). The causative fault thus exhibits an apparent listric geometry due to the coseismic interaction of shallow steep Quaternary normal faults and a deeper low-angle Miocene thrust fault reactivated with extensional kinematics (see the TDMT solution for the M4+ aftershocks in Fig. 5a,b). Our interpretation is supported by two observations. First, a relatively low-angle dipping structure was determined by both teleseismic body waves inversion (36°, see^27^) and finite-fault inversions using InSAR and GPS data (~40°; see^26,27^). Second, re-activation and inversion of the Pizzo Tre Vescovi thrust are compatible with its geometry optimally oriented in the present stress field that is characterized by NNW-oriented S_Hmax_^43^.

Differently from the Amatrice earthquake, early aftershocks of the Visso earthquake did not re-activate the MST. Only a few events fall in the N-trending cloud of off-fault aftershocks of the Amatrice earthquake showed in Fig. 2b (label *a*) and Fig. 5c (dark grey dots).

The N110° section crossing the VBFS and MST in the southern part of the activated zone (Figs 2b and 5c) is very informative. Early aftershocks of the Visso earthquake and off-fault aftershocks of the Amatrice earthquake define two distinct zones more than 3 km apart, related to the Bove segment and the eastern MST splay, respectively (cyan and dark grey dots in Fig. 5c). Within the eastern cluster, event alignment suggests a SW-dipping listric plane. Dip angle is ∼45° in the upper part (<4 km depth) and decreases progressively to ~25° below 6 km depth where the fault geometry is delineated by a narrow band of seismicity (Fig. 5c). Since field data show the low-angle geometry of the thrust planes^42^, we conclude that the thrust illuminated in the foot-wall of the VBFS should have a flat-ramp-flat geometry. The cloud of late aftershocks at the northern edge of the activated zone suggests a structure antithetic to the Cupi fault (Fig. 5a) that might relate to a Mw4.7 event occurred on November 3, 2016.

### Aftershocks of the Norcia earthquake

The Norcia mainshock ruptured the VBFS (Vettore and Bove segments, and probably also part of the Cupi fault) and the northern segment of the Laga Fault system, for a total length of ∼35 km (Fig. 3c), together with secondary faults^26,27,29^. A large part of the seismic moment was released in the top 6 km of crust by the VBFS producing a 22-km-long surface faulting^39^.

In the northernmost sector, the distribution of early aftershocks reproduces that of the Visso earthquake, and suggests re-activation of both Cupi Fault and Pizzo Tre Vescovi thrust (Fig. 2b,c). Conversely, in the central-eastern sector aftershock production is significantly higher than that of the Visso mainshock (Fig. 2b,c). Here, the Norcia early aftershocks fill a shallow gap (<3 km depth) bounded by early aftershocks of the Visso earthquake to the west and late aftershocks of the Amatrice earthquake to the east (compare Fig. 5b,d). Two distinct clusters are evident in map view (Fig. 2c). The western one elongates NNW-SSE following the Bove segment (Fig. 2c) and tends to align on a SW-dipping plane that is in agreement with the TDMT nodal plane tied to the mainshock hypocenter and with the surface ruptures (Fig. 5d). The eastern one elongates N-S in the VBFS footwall consistently with thrusts orientation (Figs 2c and 5d), thereby indicating again their re-activation.

Aftershock production is weak to the east of the mainshock epicenter (Figs 2c and 5e), consistently with the presence of the zone of maximum slip^29^, and no clear alignment of early aftershocks appears underneath the Vettore fault. The absence of aftershock alignment might be also an effect of the complexity of the deformation zone that is 2.5 km wide near the Castelluccio basin^39^.

A concentration of early aftershocks is evident at the southern edge of the activated zone (Fig. 2c). Distribution of hypocenters and TDMT solution of M4+ shallow events show that the southern aftershocks mostly relate to a structure antithetic to the Vettore and Laga Fault segments and dipping ∼60°NE under the ridge separating the Castelluccio and Norcia basins (Fig. 5e and Supporting Video S3). The remaining aftershocks correlates to the northern segment of the Laga Fault system associated with a relatively small patch of slip of the Norcia earthquake^29^. The strike and length of the antithetic fault inferred from event alignment are ∼N345° and ∼12 km, respectively. Its location is 3–4 km eastward of the antithetic fault activated by the Amatrice Mw5.4 aftershock (Fig. 2a–c).

Figure 5f shows the N120° section *L1-L2* that crosses the lateral ramp of the MST southward of the tip of the Vettore Fault (Fig. 2c). Since the section runs orthogonal to the MST and nearly parallel to the VBFS and we projected only aftershocks within 1.5 km distance, aftershocks related to the MST should cluster into a narrow band around it, whereas aftershocks related to the VBFS and antithetic faults should be smeared out over a wide area. Taken together, aftershocks of the Amatrice and Norcia earthquakes define a structure dipping WNW between 1–9 km depth eastward of Norcia mainshock hypocenter. Dip angle decreases gradually with depth from ∼45° to ∼25°. The lower part tends to merge into the deep band of seismicity that gently dips SE, while its surface projection is coherent with the trace of the MST. As expected, seismicity above the WNW-dipping structure spreads over a wide area. These findings point to re-activation of the lateral ramp of MST where it dips WNW in the hanging-wall of the VBFS. The extensional re-activation of the thrust-fault ramp is coherent with TDMT solutions of many M > 3 aftershocks showing N to NE striking nodal planes and extensional kinematics^29^ (Fig. 5f).

### Deep aftershocks

Previous studies^30^ related the deep band of aftershocks between 8–10 km depth to a strong rheological discontinuity between the Triassic evaporites (dolostones and anhydrites) of the Umbria-Marche multilayer and the phyllitic upper part of the underlying Permo-Triassic basement. Such deep band of seismicity was ascribed to a regional shear zone and its bottom defines the brittle-ductile transition^22,24^.

Our high-resolution locations give a new refined image of the deep seismicity band. Cross-sections orthogonal to the MST suggest an eastward rapid deepening of the seismicity band where it intercepts the low-dipping part of the thrust (Fig. 5f). Focal mechanisms of M4+ events are extensional with one nodal plane dipping at low angle (<30°) to the west (Fig. 5d,e). Figure 3b shows that the aftershock band of the Visso earthquake in the NW sector (cyan dots, label *b*) is shallower than that of the Amatrice earthquake in the SE sector (dark grey dots, label *b*). A dense cloud of aftershocks of the Amatrice earthquake dips NW delineating the deep part of the lateral ramp of the MST (dark grey dots, label *a*). Remarkably, the bottom of the seismicity band deepens abruptly from 8 km to 11 km depth just at the intersection with this ramp. In addition, the up-thrown sector dips clearly to northwest. All these geometries indicate a crustal-scale contractional nature of the structure illuminated by the NW-dipping cloud of aftershocks (i.e., the MST) that appears also rooted in the underlying basement, as suggested by aftershocks of the Norcia earthquake (light grey and orange dots in Fig. 3b,c, respectively).

## Discussion

### Inferences on rupture process and multi-fault rupture models

The new accurate hypocentral locations allow investigating the relationship between the deep seismicity and the normal faults ruptured by mainshocks. The activation of the deep band of seismicity follows always the mainshock and its along-strike extent, defined by M ≥ 3 early aftershocks, mimics the total length of the broken fault segments (Fig. 3a–c). In cross sections, deep early aftershocks concentrate to the west of the mainshock fault, while the number and magnitude of events increase with the mainshock magnitude (compare Figs 4a, 5b,e). These observations suggest that a link exists between deep seismicity and rupture process in the overlying block. Coulomb static stress changes induced by the Norcia mainshock allow explaining the distribution of deep aftershocks. The dense cluster of deep early aftershocks located to the west of the ruptured fault falls inside a lobe of positive Coulomb stress change (ΔCFS ∼5 bar, Supplementary Fig. S4) for receiver normal faults striking NNW and gently dipping WSW in agreement with the TDMT low-angle solutions (Fig. 5d,e). Under these conditions, slip on low-angle normal faults may be favored by the presence of: *(i)* low friction phyllites, *(ii)* high pore fluid pressure zones just below and/or within the base of the Triassic evaporites, where fluid overpressure can occur within dolostone sealed by anhydrite beds^18,44^. Since the deep discontinuity possibly separates normal friction carbonate rocks (μ = 0.6–0.8) and low friction basement phyllites^45^, it could correspond to a zone of strain rate gradients and stress accumulation where seismicity concentrates.

The aftershocks pattern showed in Fig. 3 strongly support the conclusion of^24^ of a dual role played by the MST lateral ramp in controlling rupture propagation. Early aftershocks of the Amatrice and Visso earthquakes define two distinct contiguous zones of the VBFS separated by the deep part of the MST cross-structure that was illuminated by a wealth of aftershocks of the Amatrice earthquake (dark grey dots, Fig. 3b), whereas it remained silent after the Visso earthquake. Remarkably, the Norcia earthquake nucleated just at the junction of the two aftershock zones, at the intersection with the MST cross-fault (Fig. 3c). In this view, the MST thrust ramp acted both as a structural barrier to slip propagation for the Amatrice and Visso earthquakes and as a zone of stress concentration that in turn ruptured during the Norcia mainshock. On October 30, rupture started just in this zone to propagate first onto the VBFS, then onto the northern segment of the Laga Fault system, and possibly onto the thrust ramp itself with initial left-lateral strike component^29^. Concentration of M4+ events further supports rupture of the lateral ramp during the Norcia mainshock (Fig. 3c).

Finite-fault inversions of the Norcia earthquake published so far rely on multi-segment rupture mechanisms. All slip models share a main patch on the central-southern segments of the VBFS and a minor one on the northernmost part of the Laga Fault system, but they differ strongly in the geometry and structural role of secondary faults. A N210°-striking cross-fault at the southern termination of the VBFS, which dips 36° in its hanging-wall was considered by^29^ (Supplementary Video S3). A seismic moment equivalent to Mw 6.2 event is associated with this fault interpreted as the lateral ramp of the MTS^29^. A fault comparable in strike and dip characterizes as well the model of^27^ that includes in addition a fault antithetic to the VBFS. However, these authors place such a cross-fault ∼3 km northwestward of the MST and interpret it as a blind normal-oblique Quaternary fault located to the south of the Castelluccio basin^26^. Proposed two alternative scenarios including an antithetic structure or the NNE-striking cross-fault that, differently from the above rupture models, gently dips 20° northwestward.

Our aftershock locations allow placing new geometrical and kinematic constraints on secondary faults. They confirm a SSW orientation for the cross-structure corresponding to the kinematically inverted MST lateral ramp, but also delineate a ramp-flat geometry dipping ∼45° in the upper part. Therefore, our results contrast with the gently-dipping segment of^26^, while fault position and structural interpretation of^27^ should be reconsidered. We find instead a good agreement with the solution proposed by^29^ even if their fault dipping 36° to the SW has a planar geometry imposed by the inversion approach that cannot take into account the complex listric shape outlined by our aftershocks relocations. In this view, our study corroborates previous interpretation of the MST of^24^ as a flat-ramp-flat structure. Conversely, our study contrasts with the subsurface model of^30^ because it defines a gently (∼20°) dipping MST in the hanging-wall of the Vettore segment, lacks high-angle faults in the footwall of the VBFS and thereby ascribes all eastern aftershock activity to the normal fault system.

The antithetic fault delineated by aftershocks of the Norcia earthquake (Fig. 5e and Supplementary Videos S3) reinforces models that require slip on NE-dipping segments to tackle complexities of InSAR images near Norcia^26,27^. Besides, our study unravelling two distinct antithetic structures for the Amatrice and Norcia earthquakes (Fig. 2) suggests that caution must be taken when using aftershock distribution of a complex sequence to set up inversion model geometries. The position and geometry of the northeast-dipping segment of^27^ are in close agreement with the NNW-trending antithetic fault of the Norcia earthquake reported in this study, whereas we found the segment of^26^ slightly rotated (~10° anticlockwise) and shifted (~2 km) to the west. We emphasize that the rupture of the antithetic fault defined in this study during the Norcia mainshock is supported by the good agreement with the location and geometry of the coseismic faulting that affected the San Benedetto freeway tunnel^46^ (label SB in Fig. 2). In addition, the presence of sub-parallel high-angle antithetic faults to the east of the Norcia basin agrees with seismic reflection data interpretation^30^.

The relatively shallow depth of the three relocated mainshocks (4.5, 5.6 and 6.7 km, respectively) clearly indicates nucleation inside the Umbria-Marche sedimentary multilayer - specifically within the high Vp/Vs zone ascribed to high pore fluid pressure carbonates by^31^ - and not in the metamorphic basement, as proposed at least for the Amatrice earthquake^24^. Since significantly deeper nucleation points were also used in finite-fault inversions of the Amatrice earthquake (6.5 km depth)^22^ and Norcia earthquake (9.5 km depth)^29^, we highlight that our relocated hypocenters better agree with slip patches confined to the top 5–6 km of the crust showed by all rupture models^23,24,28,29^. In the perspective of a more reliable earthquake source investigation, the improvement in the location of nucleation points of major shocks we have provided in this work should be taken into account in the future finite-fault source modeling attempts.

## Conclusion

Aftershock patterns illustrated in this study highlight the complex interaction between normal-fault systems and inherited thrusts activated during the AVN sequence and provide new insight into the multi-segment rupture mechanisms. We document reactivation of a flat-ramp structure referable to the MST. Both its N-trending frontal ramp in the VBFS footwall and its SSW-trending lateral ramp in the Vettore fault hangingwall are prolific in aftershock production. The off-fault aftershock cloud that elongates N-S following the MST frontal ramp can be explained by the Coulomb static stress changes induced by the Amatrice earthquake^47^, whereas aftershock activity along the MST lateral ramp supports the rupture model of the Norcia earthquake of^29^ that requires significant slip on this cross-structure. The spatial partitioning of early aftershocks evidences the dual control played by the MST lateral ramp on the rupture propagation (barrier and asperity) in agreement with interpretation of^24^. We hypothesize re-activation and kinematic inversion also for the optimally oriented Pizzo Tre Vescovi thrust whose deep part was likely ruptured by the Visso earthquake. Two distinct main antithetic faults ruptured during the August 24 Mw5.4 aftershock and the Norcia mainshock, respectively. Therefore, our study suggests to consider both the MST cross-structure and the eastern antithetic fault in future finite-fault source modeling of the Norcia earthquake that hopefully should combine strong motion, GPS and InSAR data.

Our high-quality manually reviewed catalog is of key importance for future seismological applications. For a prolific aftershock sequence like AVN, a high-quality initial catalog with a wide range of magnitude is a key ingredient for the successful application of detection techniques based on template waveforms cross-correlation^48^ and for optimizing automated picking algorithms^49^. Then, application of high-precision double-difference relative location techniques strictly depends on the quality of the earthquake catalog and on the accuracy of initial absolute locations^50^. Future finite-fault inversions will benefit from geometric constraints on the activated fault systems and mainshock locations provided by our study. The new high-quality hypocentral locations will allow improving comparison between structures imaged by seismic reflection data and aftershock distribution.

## Methods

We located both aftershocks and mainshocks using the inversion program NonLinLoc (hereafter called NLL)^33^, which is based on the probabilistic approach proposed by^51^ and on a robust non-linear inversion scheme. NLL offers the advantage to provide a comprehensive estimate of the location uncertainties through the construction of the *a posteriori* probability density function (PDF). Following the probabilistic approach, the optimal earthquake hypocenter location is represented by the maximum likelihood point of the computed PDF, while the shape and the size of the PDF are representative of the location uncertainty (see Supplementary Materials).

The NLL inversion grid extends over an area of 120 × 120 km^2^, centered on the epicenter of the Norcia mainshock (Fig. 1). We used only 59 stations falling in this area to avoid traveltime errors due to the 1-D model approximation at the Moho depth. Around 110,600 P and 98,100 S first arrival traveltimes were computed in a medium obtained by smoothing Vp and Vs minimum 1-D models determined for the Umbria-Marche Apennines^52^ (Fig. S5). Station correction terms were included in the inversion procedure to compensate approximately for 3-D velocity heterogeneities. Station corrections have a distribution consistent with the upper crustal structure and show large positive values (up to 2.0 s both for P- and S-data) in the easternmost region characterized by low-velocity (Vp < 3 km/s) Plio-Pleistocene foredeep sediments up to 4–5 km thick. Distribution of location quality parameters are showed and discussed in the Supplementary Fig. S2.

## Supplementary information

Supplementary Materials

Supplementary Video S1

Supplementary Video S2

Supplementary Video S3

Data S1

## Data Availability

Waveform data of earthquakes recorded by the INGV stations are available from the European Integrated Data Archive (EIDA) at http://eida.rm.ingv.it. P- and S-phase arrival times of the early aftershocks analyzed in this study are available in the database ISIDe (Italian Seismological Instrumental and parametric Data-base; http://cnt.rm.ingv.it/iside). The catalog of early aftershocks published in this study is available in the Supplementary Data File S1 (file DataS1_EAcatalog.docx uploaded separately).
