# Supplementary material for: Multi-segment rupture of the 2016 Amatrice-Visso-Norcia seismic sequence (central Italy) constrained by the first high-quality catalog of Early Aftershocks
Source: Sci Rep. 2019 May 6;9:6921. doi: 10.1038/s41598-019-43393-2 (PMC6502830; doi:10.1038/s41598-019-43393-2)
Supplement: Supplementary file 1 — Supplementary Materials [file 41598_2019_43393_MOESM1_ESM.pdf]

Supplementary Materials for

Multi-segment rupture of the 2016 Amatrice-Visso-Norcia seismic sequence (central Italy) constrained by the first high-quality catalog of Early Aftershocks

Luigi Improta[1], Diana Latorre[1], Lucia Margheriti[1], Anna Nardi[1], Alessandro Marchetti[1], Anna Maria Lombardi[2], Barbara Castello[1], Fabio Villani[2], Maria Grazia Ciaccio[2], Francesco Mariano Mele[1], Milena Moretti[1], and the Bollettino Sismico Italiano Working Group[#]*

Affiliation and address:

[1]Istituto Nazionale di Geofisica e Vulcanologia, Osservatorio Nazionale Terremoti, Roma, Italy
[2]Istituto Nazionale di Geofisica e Vulcanologia, Sezione Roma 1, Roma, Italy

[#]A full list of members with affiliations appears in the Author Contributions

Correspondence to:

luigi.improta@ingv.it (Luigi Improta)

Analysis of completeness magnitude

Reasons why not all earthquakes of a sequence are detected are multifold: (1) some events are too small to be detected within the coda of larger events (i.e. increased noise), (2) operators decided that events below a certain threshold are not of interest and (3) the occurrence frequency of events is too high to be followed in real time.

The main topic of present study is to recognize the complex architecture of faults activated by the Amatrice-Visso-Norcia sequence, by earthquake locations of a high-quality catalog. Assessing the magnitude of completeness (*Mc*) of the catalog is not an essential step for this type of analysis. In any case. we decided to briefly estimate *Mc* to quantify the improvement of data after our careful revision of the earthquakes detected by the INGV real-time monitoring system. Here we only report completeness magnitudes and the b-values (*b*) of the Gutenberg-Richter law, for both the new catalog (hereinafter Rev) and the one that contains aftershocks revised routinely in quasi-real time at the INGV surveillance center that were used by Chiaraluce et al. (2017) (hereinafter NRev). We considered events occurred in the first 24 hours after the Amatrice (2016 Aug 24, 01:36:32 UTC, Mw6.0), the Visso (2016 Oct 26, 17:10:36 UTC, Mw5.9) and the Norcia (2016 Oct 30, 07:40:17 UTC, Mw6.5) earthquakes.

We estimate *Mc* by applying the Goodness of Fit method (Woessner and Wiemer, 2005), based on the maximum likelihood estimation of b-value, measuring the probability *prMc* that the Gutenberg-Richter law explains the data above *Mc*. We select *Mc* as the lowest magnitude for which *prMc* ≥ 95%. If *prMc* is systematically lower than 95%, we select the magnitude maximizing it.

Results are listed in the table showed below and shown in Figure S1. The worst completeness is found after the Norcia event, for both NRev and Rev datasets, for which the probability threshold of 95% is not reached, not even after revising data. This event occurred after more than two months from the beginning of the sequence, when the temporary network had been fully deployed in the epicentral area. The worst *Mc* is, therefore, imputable to large magnitude of Norcia event, causing a larger increase of noise, respect to other strong events.

	**Amatrice**		**Visso**		**Norcia**	
	**NRev 395 events**	**Rev 1568 events**	**NRev 480 events**	**Rev 1363 events**	**NRev 506 events**	**Rev 1481 events**
**Mc**	2.7	2.2	2.4	2.2	3.4	2.9
**b**	0.8	0.9	0.9	0.9	1.2	1.0
**prMc**	94%	95%	92%	95%	94%	93%

Our revision increases the number of events by roughly threefold in the three selected time windows (the Rev datasets are 2.8 to 3.9 times larger depending on the mainshock). All estimated b-values have an error equal to about 0.1 and, therefore, do not show significant changes in time.

A previous estimation of *Mc* was done soon after the start of the sequence (Marchetti et al., 2016), giving *Mc* =3.4 and *Mc*=2.7 for the first 8 hours after the Amatrice event, for NRev and Rev datasets, respectively. By considering the strong temporal variation of detection after a strong event, these values are in agreement with results of present study.

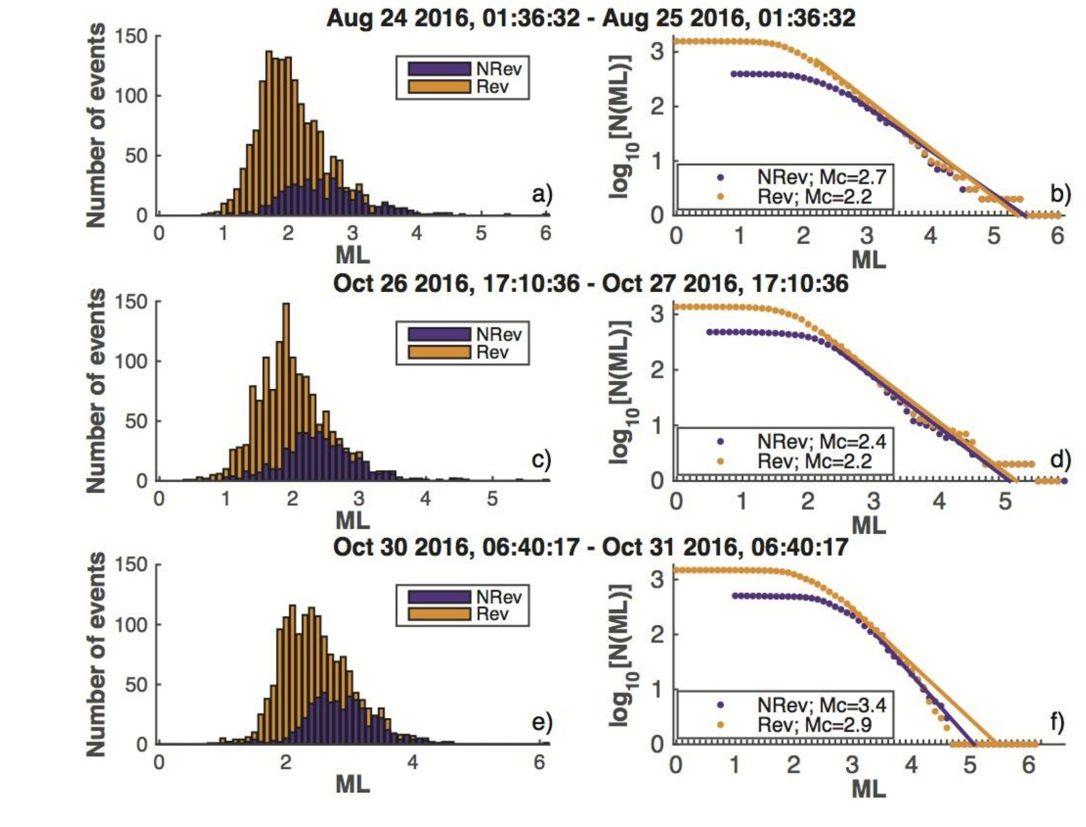

Figure S1. (left) Magnitude distribution histograms of the catalogues containing aftershocks analyzed in this study (Rev) and revised routinely in quasi-real time at the INGV surveillance center (NRev). (right) Frequency-magnitude distribution of the Rev and NRev catalogues. The continuous lines indicate the fit of the Gutenberg-Richter model. The completeness magnitude (Mc) is also reported.

Aftershocks locations: quality parameters of the new catalog and comparison with event locations determined by using data analyzed in quasi real-time at the INGV surveillance center

Early aftershocks were located using the inversion program NonLinLoc (NLL, Lomax et al., 2000; Lomax et al., 2009), which is based on the probabilistic approach proposed by Tarantola and Vallette (1982) and a robust non-linear inversion scheme. NLL offers the advantage to provide a comprehensive estimate of the location uncertainties through the construction of the a posteriori probability density function (PDF). Following the probabilistic approach, the optimal earthquake hypocenter location is represented by the maximum likelihood point of the computed PDF, while the shape and the size of the PDF are representative of the location uncertainty.

Our analysis of waveforms recorded by all permanent and temporary stations has led to producing a new dataset characterized by a greatly improved number of P and S phase picks, as well as by a more rigorous and coherent evaluation of their picking quality with respect to data used in previous studies (e.g., Chiaraluce et al., 2017) that were revised routinely in quasi real-time by seismologists on duty at the INGV surveillance center.

Figure S2 shows the distribution of location quality parameters of the new catalog. About 80% of the earthquakes has good-quality location parameters: number of phases ≥ 22, *rms* (root-mean-square residuals) ≤ 0.12 s, azimuthal gap ≤ 89°, epicentral distance to the closest station ≤ 9 km, horizontal formal location error (2σ) ≤ 0.6 km, vertical formal location error (2σ) ≤ 2.0 km, PDF volume ≤ 0.8 km^3 (see Lomax et al., 2009). The median values of parameters distribution are as follow: number of phases = 35, *rms* = 0.09 s, azimuthal gap = 60°, epicentral distance to the closest station = 5 km, horizontal formal location errors = 0.4 km, vertical formal location errors = 1.1 km, PDF volume = 0.29 km^3.

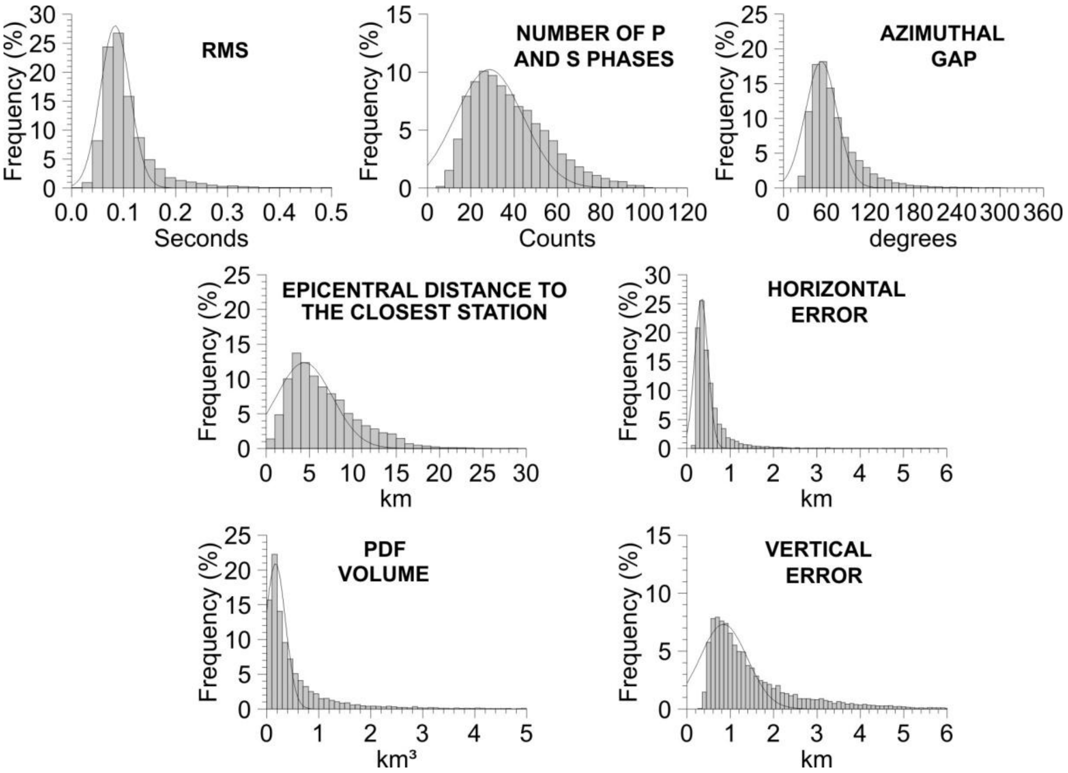

Figure S2. Distribution of location quality parameters of the catalog presented in this study.

In order to show the impact of the improved data quality on the hypocenter location estimation, we compared location parameters for 3,400 early aftershocks that were firstly revised in quasi real-time at the INGV surveillance center and later analyzed in this study. For this test, our hand-picked data and those revised at the INGV surveillance center, which were used by Chiaraluce at al. (2017), were inverted applying the NLL algorithm with the same inversion parameter tuning and velocity model.

The main parameters that define the location quality are compared in the panels of Figure S3, where data obtained by inverting our hand-picked arrival times (hereinafter Revised Data, horizontal axis) are plotted against data obtained by inverting the arrival times analyzed at the INGV surveillance center (hereinafter Seismic Room Data, vertical axis). In addition, the histograms included in each panel help us to analyze the frequency distribution of the location

parameters obtained from the inversion of arrival times read in this study or analyzed at the INGV surveillance center (white-blue and red bins, respectively).

The two datasets mainly differ in the number of P- and S-phase first arrivals used to locate each earthquake (panel a) that significantly increased after our revision from an average value of ~25 for the Seismic Room Data to ~50 for the Revised Data.

The comparison of the *rms* values (panel b) shows that observed arrival times of both datasets are well reproduced, as indicated by the similar histograms and the low *rms* values (mostly < 0.2 s). However, if we consider the distribution of the *rms* values with respect to the number of phases used in the hypocenter location (panel c), we note that the dependence of the *rms* parameter on the number of phases is very strong for the Seismic Room Data (red dots). Indeed, very-low *rms* values (< 0.1 s) are related to a reduced number of data used in the inversion procedure (phases < 30). Differently, earthquake locations determined by using the Revised Data (blue dots) have low *rms* values even in case of a wealth of inverted data.

The remarkable addition of arrival time picks in the Revised Data has the effect of strongly reducing the azimuthal gap (panel d) and the epicentral distance to the closest station (panel e). This two parameters control the horizontal error (panel f) and the vertical error (panel e), respectively. In particular we observe that the percent of locations having azimuthal gap < 60° increases from 32% to 73%, while the percent of locations having the epicentral distance to the closest station < 5 km increases from 27% to 53%. This last point is of key importance to obtain well resolved hypocenter locations also for shallow aftershocks.

The increased number of phases used in the inversion procedure, combined with the reduction of both the azimuthal gap and the epicentral distance to the closest station, have a strong impact on the uncertainty of the location results in terms of standard error and resolution (panels f-i). This improvement is evident by comparing the value of two parameters that describe the uncertainty of the hypocenter location for the two dataset. The first parameter is the volume of the PDF that is reconstructed by the NLL inversion (panel h). Well constrained hypocenter locations are characterized by small PDF volumes. By comparing the inversion results of the two datasets we clearly observe that the Revised Data provide solutions characterized by a remarkable reduction of the PDF volume. Seismic Room Data provide indeed a high number of hypocenter locations with large uncertainty. The percent of locations having PDF volumes < 1 km^3 increases from 36% to 75%. The second parameter indicating the location uncertainty is the distance between the maximum likelihood point of the computed PDF and the expectation hypocenter location, i.e., the Gaussian statistic hypocenter location (Lomax et al., 2000). In simplified cases, when the hypocenter location problem is well constrained by the available data the PDF forms a near-ellipsoidal shape and contains a single maximum likelihood point. In this case, the likelihood hypocenter location coincides to the Gaussian statistic hypocenter location. Conversely, when the hypocenter location problem is not well constrained, the PDF representation can have irregular form with multiple, local maximum likelihood points. In this second case, the distance between the maximum likelihood hypocenter location and the expectation location indicates the irregular and multimodal character of the PDF and therefore, a poor constrained hypocenter location. Looking at the Figure S3 (panel i), we can note that hypocenter solutions of the Seismic Room Data are often affected by irregular PDF with respect to the solutions of the Revised Data that are mostly very well constrained. The remarkable improvement in earthquake location accuracy is evident when comparing the plots of the horizontal and vertical formal errors (panel f and g, respectively). The

percent of locations having horizontal formal location errors < 0.6 km increases from 36% to 78%, while for vertical formal location errors < 1.5 km the percent increases from 27% to 71%.

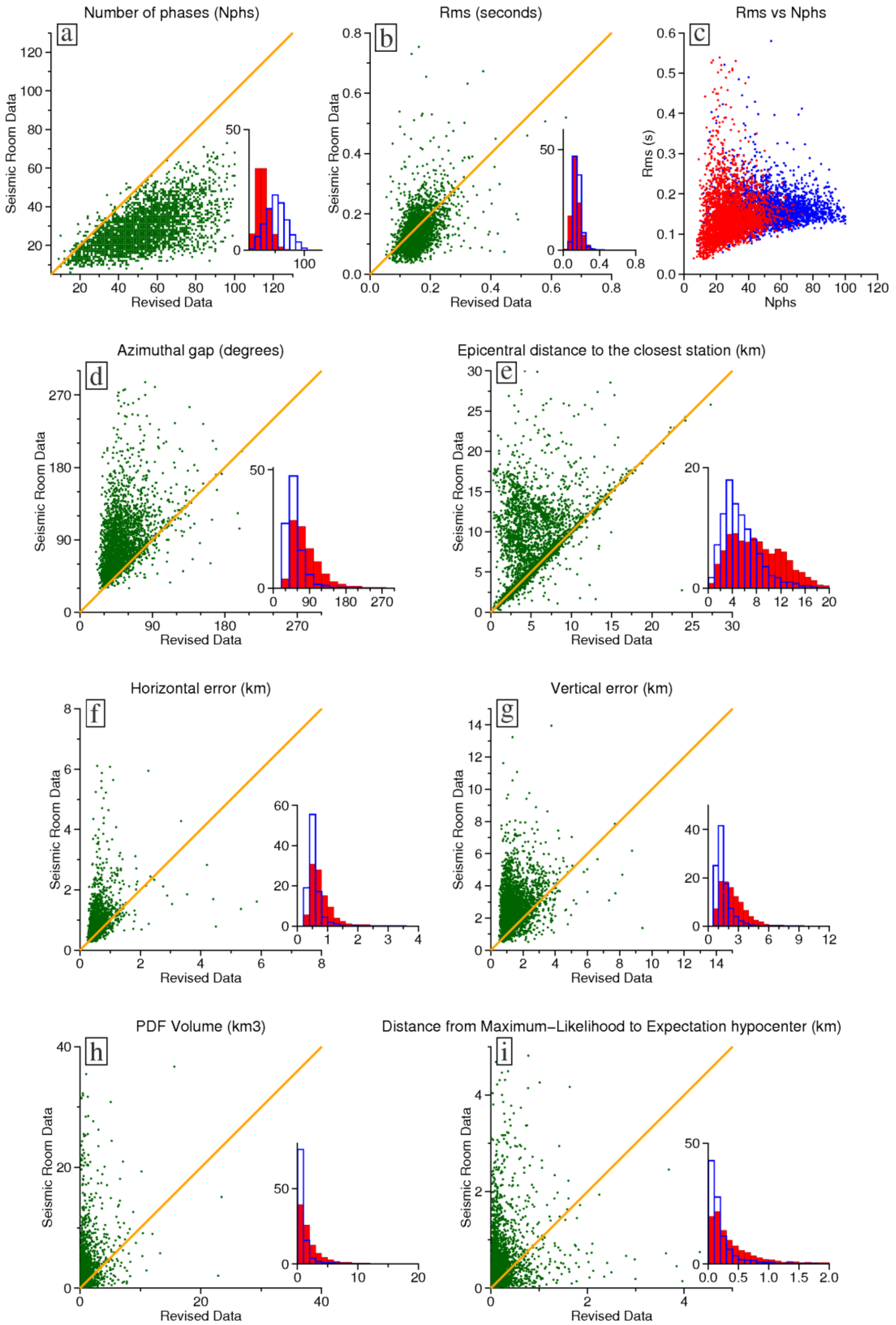

Figure S3. Comparison of location parameters for 3,400 early aftershocks obtained by inverting arrival time data revised in quasi real-time at the INGV surveillance center (Seismic Room Data in the plot) and arrival time data handpicked in this study (Revised Data in the plot). The histograms show the frequency distribution of the location parameters obtained from the inversion of arrival times read in this study or analyzed at the INGV surveillance center (white-blue and red bins, respectively).

Coulomb Static Stress Change

To understand the nature of the high aftershock production within the deep band of seismicity to the west of the mainshock sources, we investigated whether the distribution of early aftershocks is compatible with the elastic Coulomb stress transfer due to the 30 November, Mw6.5, Norcia earthquake.

The induced Coulomb stress change field ΔCFS can be represented by:

$$\Delta CFS = \Delta\tau + \mu\Delta\sigma$$

where $\Delta\tau$ is the shear stress change, μ is the frictional coefficient and $\Delta\sigma$ is the normal stress change.

The Coulomb stress change was computed by using Coulomb 3.3 software (Toda et al., 2011). In this test we used the planar fault of the Norcia earthquake as source, and considered receiver faults with a mechanism similar to that of the TDMT solutions of the M4+ deep early aftershocks (i.e., NNW-striking normal-faulting mechanism with one nodal plane dipping at low-angle to the WSW) (http://cnt.rm.ingv.it/tdmt). For the Norcia mainshock we used the mechanism reported by Scognamiglio et al. (2018) (strike = N155°, dip = 47°, rake = -90°), for the receiver faults we used the following strike, dip and rake: N155°, 20°, -90°. The frictional coefficient μ is set to be 0.6.

The position of the causative fault is constrained by the Mt. Bove - Mt. Vettore primary coseismic ruptures (Villani et al., 2018) (Fig. S4a) and by the mainshock hypocenter determined in this study (Fig. S4c). The source dimension (35 km along strike and 12 km along dip) is consistent with finite-fault kinematic inversion of Scognamiglio et al. (2018). The top of the planar fault is at 1 km above sea level (i.e. the average elevation in the region) and matches primary co-seismic ruptures.

The computed Coulomb stress transfer is compared with the distribution of early aftershocks of the Norcia earthquake (Fig. S4a) along a vertical cross-section perpendicular to the source direction (section A-B in Fig. S4b). Section A-B matches the profile H1-H2 (Fig. 3c) selected to plot the depth distribution of the early aftershocks.

Figure S4c shows that the pattern of the Coulomb stress change is quite complex in correspondence of the lower edge of the causative fault. Between 8 and 10 km depth (i.e., the depth range of the deep band of seismicity), two positive lobes are separated by a narrow vertical zone with negative Coulomb stress change (-5 bar) located just beneath the tip of the fault. The increase in Coulomb stress is higher for the western lobe (5 bar) that dips gently westward, with respect to the eastern one that dips steeply eastward (3 bar).

Figure 4d shows that the dense cluster of deep early aftershocks located to the west of the ruptured fault falls inside the western lobe of positive Coulomb stress change and matches the zone of maximum increase. In addition, the cluster is bounded to the east by the vertical zone of negative stress change.

Aftershocks do not deepen westward following the shape of the positive western lobe. Indeed, the deep seismicity relates to a strong, sub-horizontal rheological discontinuity between the Triassic evaporites (dolostones and anhydrites) of the Umbria-Marche multilayer and the phyllitic upper part

of the underlying Permo-Triassic basement. Such a discontinuity behaves as a regional shear zone and its bottom defines the brittle-ductile transition (Pizzi et al., 2017).

A second test was performed by considering as receiver faults those dipping at high angle to the ENE (strike = N335°, dip = 70° and rake = -90°). The comparison of the computed Coulomb stress changes along the vertical cross-section A-B evidences an anticlockwise rotation of the two positive lobes and the disappearance of the vertical zone of pronounced stress decrease. Such a pattern of Coulomb stress change cannot explain the observed aftershock distribution.

These results indicate that the re-distribution of static stress after the Norcia earthquake allows explaining the burst of off-fault deep aftershocks to the west of the mainshock hypocenter, if we assume normal-faulting mechanisms striking NNW and gently dipping WSW in agreement with TDMT solutions of M4+ events.

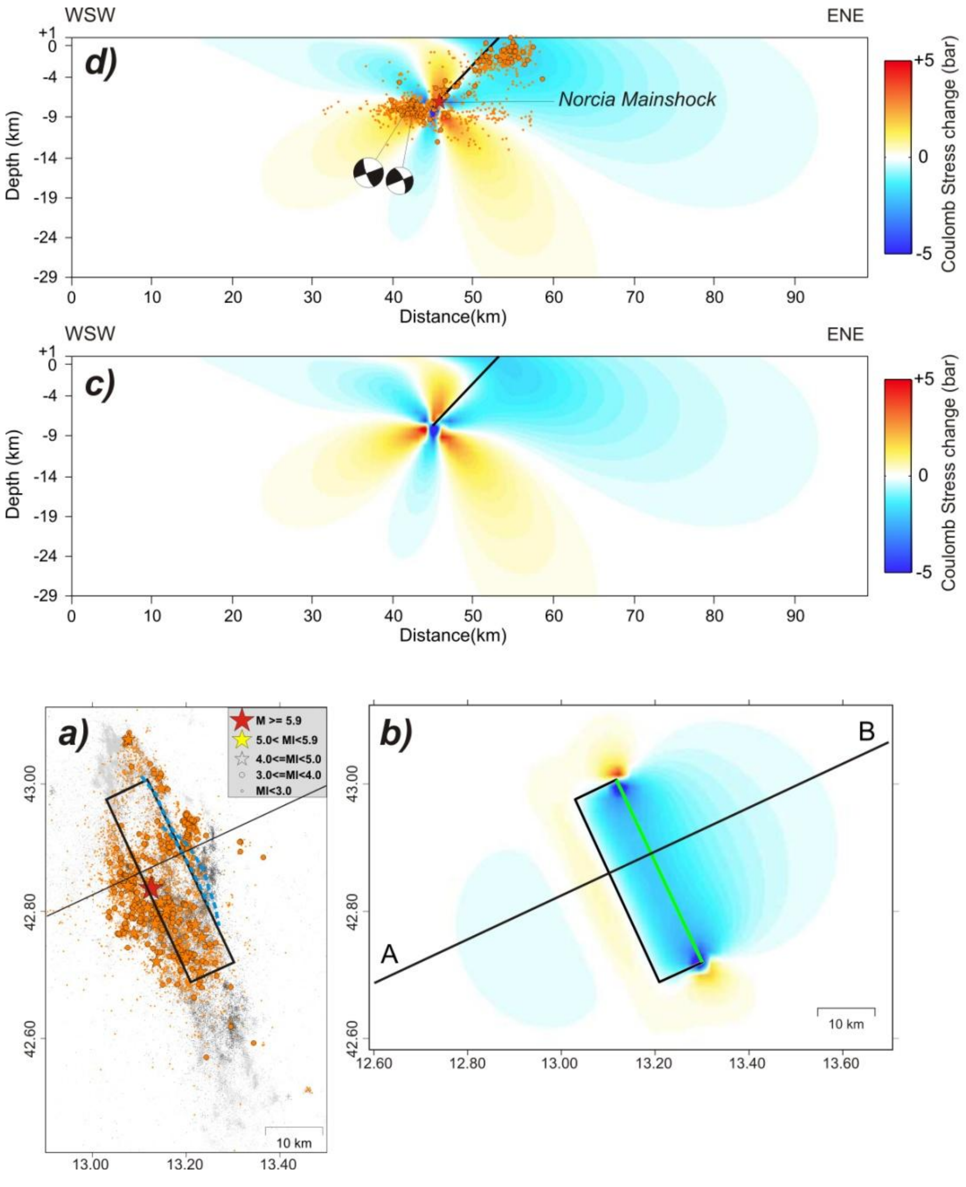

Figure S4: Comparison between Coulomb stress transfer computed for the 30 October 2016, M_w 6.5, Norcia earthquake and the distribution of early aftershocks along a vertical cross-section perpendicular to the source direction. (a) Map view of early aftershocks of the Norcia earthquake (orange symbols). The blue dashed lines define schematically coseismic surface ruptures associated

to the Vettore-Bove fault system. The black rectangle identifies the surface projection of the modelled source, the black line the trace of the vertical section A-B. The red star denotes the mainshock epicenter. (b) Coulomb stress transfer field (in bar) computed at the top of the model. The black rectangle identifies the surface projection of the source and the black line the trace of the vertical section A-B. The green line corresponds to the fault trace at surface. (c) Cross-section of Coulomb stress transfer along the profile A-B. The continuous black line corresponds the intersection of the cross-section with the fault plane. (d) Early aftershocks are projected onto the Coulomb stress transfer cross-section (the plotted hypocenters are within 3 km distance from the section). Focal mechanisms are projected TDMT solutions of two M4+ events.

Aftershocks locations: 1-D Velocity model

P- and S-phase first arrival traveltimes were computed in a 1-D local velocity model. The model was obtained by smoothing the Vp and Vs minimum 1-D models determined for the Umbria-Marche Apennines in a previous tomographic study by Carannante et al. (2013). The Figure S5 shows the Vp, Vs and Vp/Vs models used in this study to locate the aftershocks and mainshocks. High Vp and Vp/Vs values in the upper 4 km of the crust (Vp=5.6-6.0 km/s and Vp/Vs=1.90-2.05) can be related to fractured, high pore fluid pressure Mesozoic carbonates of the Umbria-Marche multilayer. High Vp values and ordinary Vp/Vs values between 4 and 20 km depth (Vp~6.2 km/s and Vp/Vs~1.8) can be associated with Triassic evaporites (mainly dolostones) in the lower part of the Umbria-Marche sedimentary sequence and with the underlying crystalline basement.

.

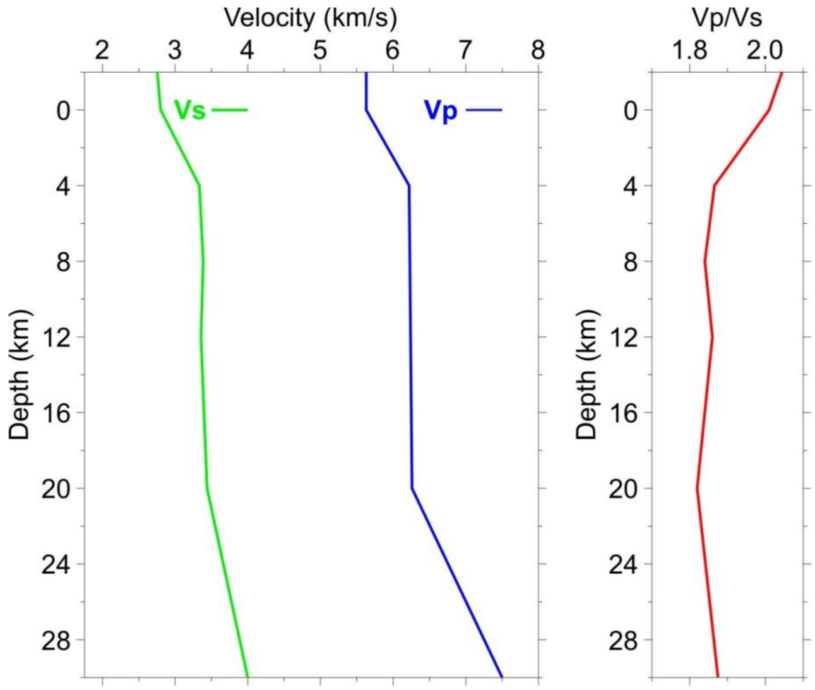

Figure S5: P and S wave 1-D velocity models used in this study to locate the aftershocks and mainshocks of the Amatrice-Visso-Norcia sequence. The figure also shows the corresponding Vp/Vs ratio.

Re-location of the mainshocks and major aftershocks

We carefully revised strong motion recordings of permanent and temporary INGV stations, complemented by 11 to 19 stations of the Accelerometric National Network run by Department of Civil Protection. With respect to previous catalogues, our re-location provides significantly shallower hypocentral depth (depth difference >1 km) for all events but the Norcia mainshock (see Supplementary Table S1 online). Table S1 reports hypocenter parameters of the mainshocks and major aftershocks re-located in this study. We remark that both the Visso earthquake and its strong foreshock are double events.

Date(yr-m-d)	Time (UTC)	Longitude	Latitude	Depth (km)	Mw
2016-08-24	01:36:32.87	13.2491°E	42.7013°N	4.55	6.02
2016-08-24	02:33:29.84	13.1610°E	42.7971°N	7.33	5.39
2016-10-26<br>2016-10-26	17:10:37.16<br>17:10:37.93	13.1366°E<br>13.1254°E	42.8825°N<br>42.8720°N	4.24<br>2.51	4.54<br>5.39
2016-10-26<br>2016-10-26	19:18:06.76<br>19:18:08.25	13.1385°E<br>13.0958°E	42.9058°N<br>42.9052°N	3.68<br>5.67	4.50<br>5.83
2016-10-30	06:40:18.13	13.1247°E	42.8358°N	6.75	6.50

Table S1. Hypocenter parameters of the mainshocks and major aftershocks re-located in this study.

Catalog of Early Aftershocks

The catalog of early aftershocks published in this study is available in the Supplementary Data file S1 (file DataS1_EAcatalog.docx uploaded separately). The catalog reports the hypocentral parameters, location errors and local magnitude of the 10,574 early aftershocks. The map and section in the main text show only aftershocks having vertical location errors < 2.5 km.

3D Animation of the Early Aftershocks

The 3D representation of the early aftershocks aids in the understanding the spatial distribution of earthquakes and their relationship with finite-fault source models. Three videos, one for each mainshock, show: (i) the distribution of early aftershocks, (ii) the distribution of late aftershocks occurred in the time interval between the considered mainshock and the next one, (iii) the hypocenter of the mainshock, (iv) the planar source reported by other authors that performed finite-

fault inversion of the mainshock, (v) the plane of antithetic faults defined in our study through alignment of aftershocks (i.e., the antithetic faults activated by the Amatrice and Norcia earthquakes), (vi) the surface traces of the co-seismic ruptures associated with the Bove-Vettore normal-fault system, of the Mts Sibillini Thrust and of the Pizzo Tre Vescovi thrust fault.

Early aftershocks of the Amatrice, Visso and Norcia earthquakes are showed in the supplementary videos S1, S2 and S3, respectively (files VideoS1_EA_Amatrice.avi, VideoS1_EA_Visso.avi, VideoS1_EA_Norcia.avi, uploaded separately).

We prepared the movies using the Paraview software v 5.5.2 (http://www.paraview.org/) and they can been displayed using any application supporting .avi files. They are 100 s long each (1 frame/s), and show the investigated volume through an orbital rotation with a focal point centered at UTM coordinates (zone 33N) E344594 m N4743440 m and -7968 m depth, and a view angle of 30°.

Supplementary Video S1 (file VideoS1_EA_Amatrice.avi)

The video shows a 3-D perspective of the early aftershocks of the Amatrice earthquake (period 2016/08/24 to 2016/08/26). The pale yellow rectangle is the mainshock source of the Amatrice earthquake reported by Tinti et al. (2016), while the pale orange rectangle is the subsidiary antithetic normal fault related to its major aftershock we propose in this study based on the alignment of relocated early aftershocks. The small yellow spheres are the early aftershocks of the Amatrice earthquake (the mainshock hypocenter is represented with a greater sized sphere), while the small white points are the late aftershocks recorded until 26 October 2016. The red lines are the coseismic surface ruptures of the Norcia, Visso and Amatrice earthquakes (after Villani et al., 2018), the blue thick line is the surface trace of the Mts Sibillini Thrust and the purple thick line is the surface trace of the Pizzo Tre Vescovi Thrust.

Supplementary Video S2 (file VideoS2_EA_Visso.avi)

3-D perspective of the early aftershocks of the Visso earthquake (period 2016/10/26 to 2016/10/27). The pale green rectangle is the mainshock source of the Visso mainshock (after Chiaraluce et al., 2017). The small green spheres are the early aftershocks of the Visso earthquake (the mainshock hypocentre is represented with a greater sized sphere), while the small white points are the late aftershocks recorded until 30 October 2016. The large yellow sphere denotes the hypocenter of the Amatrice earthquake.

Supplementary Video S3 (file VideoS3_EA_Norcia.avi).

3-D perspective of the early aftershocks of the Norcia earthquake (period 2016/10/30 to 2016/11/01). The two pale blue rectangles are the N155°-trending main source and the N210°-trending cross-fault of the Norcia earthquake by Scognamiglio et al. (2018). The pale yellow rectangle is the NE-dipping antithetic fault we propose in this study based on the alignment of relocated early aftershocks. The small blue spheres are the early aftershocks of the Norcia earthquake (the mainshock hypocentre is represented with a greater sized sphere), while the small white points are the late aftershocks recorded until 11 January 2017. The large yellow and green spheres denote the hypocenters of the Amatrice and Visso earthquakes, respectively.

References

Marchetti, A. et al. The Italian Seismic Bulletin: strategies, revised pickings and locations of the central Italy seismic sequence. *Annals of Geophysics*, **59**, Fast Track 5, 2016; DOI: 10.4401/ag-7169 (2016).

Woessner, J. & Wiemer, S. Assessing the quality of earthquake catalogues: Estimating the magnitude of completeness and its uncertainty. *Bulletin of the Seismological Society of America*, **95** (2), 684-698 (2005).

Lomax, A., Virieux, J., Volant, P. & Berge, C. Probabilistic earthquake location in 3D and layered models: Introduction of a Metropolis-Gibbs method and comparison with linear locations in Advances in Seismic Event Location (eds. Kluwer), 101-134 (Thurber, C.H., & Rabinowitz, N.) (2000).

Lomax, A., Michelini, A. & Curtis, A. Earthquake Location, Direct, Global-Search Methods, in *Encyclopedia of Complexity and System Science*, Part 5, Meyers, R. A. (ed.), Springer, New York, pp. 2449-2473, doi:10.1007/978-0-387-30440-3 (2009).

Tarantola, A. & Valette, B. Generalized Nonlinear Inverse Problems Solved Using the Least Squares Criterion. *Rev. Geophys. Space Phys.*, **20**, (2), 219–232, (1982).

Chiaraluce, L. et al. The 2016 central Italy seismic sequence: A first look at the mainshocks, aftershocks, and source models. *Seismol. Res. Lett.*, **88** (3), 757-771; 10.1785/0220160221 (2017).

Toda, S., Stein, R. S., Lin, J. & Sevilgen, V. Coulomb 3.3 graphic-rich deformation and stress-change software for earthquake, tectonic, and volcano research and teaching - User guide, U.S. Geological Survey Open-File Report 2011-1060, p. 63 (2011).

Scognamiglio, L. et al. Complex fault geometry and rupture dynamics of the MW 6.5, 30 October 2016, Central Italy earthquake. *J. Geophys. Res.*, **123**, 2943–2964; 10.1002/2018JB015603 (2018).

Pizzi, A., Di Domenica, A., Gallovič, F., Luzi, L. & Puglia, R. Fault segmentation as constraint to the occurrence of the main shocks of the 2016 Central Italy seismic sequence. *Tectonics*, **36**, 2370–2387; 10.1002/2017TC004652 (2017).

Carannante, S., Monachesi, G., Cattaneo, M., Amato, A. & Chiarabba, C. Deep structure and tectonics of the northern-central Apennines as seen by regional-scale tomography and 3-D located earthquakes. *J. Geophys. Res. Solid Earth*, **118**, 5391–5403; 10.1002/jgrb.50371 (2013).

Tinti, E., Scognamiglio, L., Michelini, A. & Cocco, M. Slip heterogeneity and directivity of the M_L 6.0, 2016, Amatrice earthquake estimated with rapid finite-fault inversion. *Geophys. Res. Lett.*, **43**, 10,745-10,752, 10.1002/2016GL071263 (2016).

Villani, F. et al. & the Open EMERGEO Working Group. A database of the coseismic effects following the 30 October 2016 Norcia earthquake in Central Italy. Science Data, **5**, 180049; 10.1038/sdata.2018.49 I (2018).
